# A Unique Presentation of Pulmonary Vein Thrombosis in a Patient With Rapidly Progressive Metastatic Pelvic Myxofibrosarcoma

**DOI:** 10.7759/cureus.28887

**Published:** 2022-09-07

**Authors:** Lex P Leonhardt, Aamir Pervez, Wesley Tang, Marvin Amen

**Affiliations:** 1 Internal Medicine Residency, Kettering Medical Center, Dayton, USA

**Keywords:** myxofibrosarcoma, metastatic, pulmonary vein thrombosis, anticoagulation, dyspnea, elderly, pulmonary critical care

## Abstract

Pulmonary vein thrombosis (PVT) is an uncommon but often lethal disease with non-specific manifestations. Herein, we present a case of an elderly woman with a past medical history significant for limited-stage pelvic myxofibrosarcoma (MFS), who was admitted with subacute weakness and worsening dyspnea. Over the course of hospitalization, CT angiography was performed, which demonstrated a filling defect in the inferior left pulmonary vein consistent with PVT, in addition to evidence of widespread metastatic disease. The patient was offered therapeutic anticoagulation, but given the extent of her metastasis, the patient opted to transition to comfort care and pursued hospice care. This case serves as a unique presentation of a rare disease process associated with widespread metastatic MFS, illustrating the need for early recognition and treatment.

## Introduction

Pulmonary vein thrombosis (PVT) is an uncommon but potentially lethal disease associated with a broad spectrum of conditions ranging from primary malignancy to radiofrequency ablation. This condition poses diagnostic challenges to clinicians due to non-specific presentations and the occasional need for multimodal imaging strategies for definitive diagnosis [1]. While previous case reports of PVT secondary to sarcoma, namely osteosarcoma and liposarcoma, have previously been published, herein we present a unique case of PVT secondary to high-grade, rapidly progressive, metastatic pelvic myxofibrosarcoma (MFS) in an elderly patient.

## Case presentation

A 71-year-old female with a past medical history significant for pelvic MFS status-post surgical debulking, hypertension, chronic obstructive pulmonary disease, and hypothyroidism presented to the emergency department complaining of a three-week history of generalized weakness and acutely worsening dyspnea.

**Figure 1:**
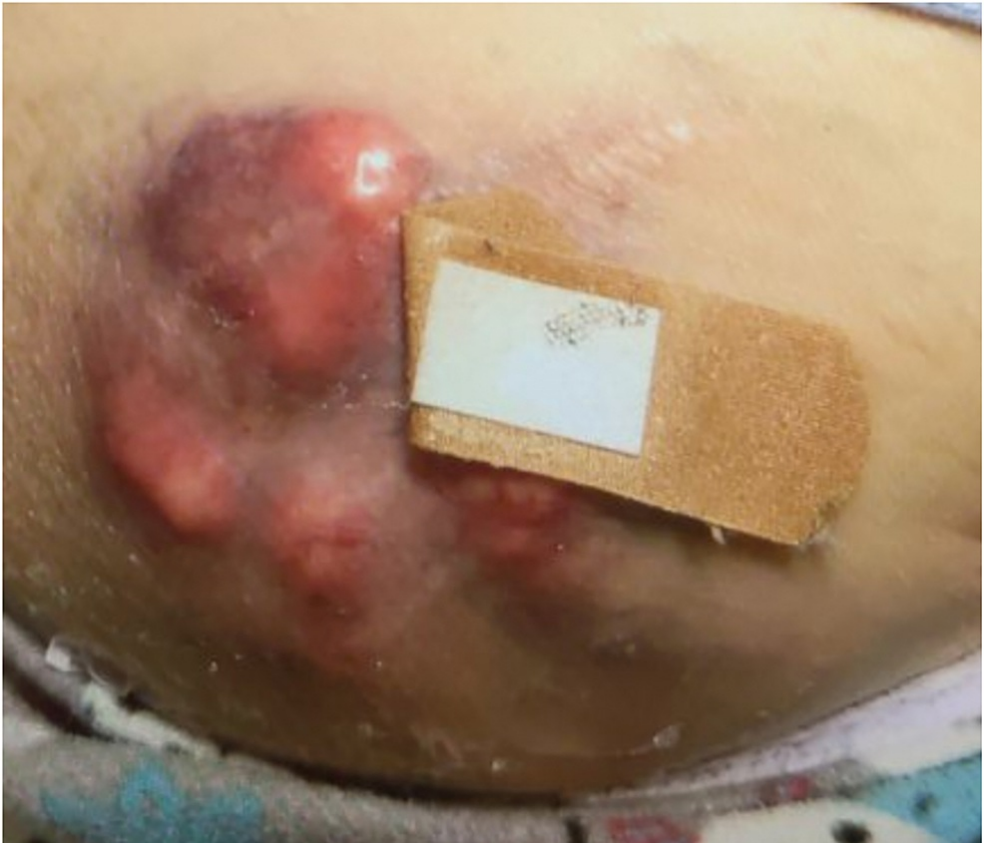
Right hip fungating mass prior to resection

Two months prior to presentation, the patient was acutely diagnosed with high-grade MFS limited to the pelvis, for which she underwent resection of soft tissue sarcoma of the pelvis with subsequent pedicled transverse rectus abdominis myocutaneous flap to repair the right hip defect (Figure 1). Surgical pathology at the time of diagnosis exhibited high-grade spindle and pleomorphic sarcoma, FNCLCC (Fédération Nationale des Centres de Lutte Contre le Cancer) grade 3, measuring 14.8 x 10.5 x 9.3 cm with invasion into the skeletal muscle, dermis, and lymphovascular supply. Surgical margins were negative with no invasion into the deep fascia/dermis. CT imaging of the chest performed prior to operative resection of the tumor did not demonstrate suspicious pulmonary nodules or lymphadenopathy. Radiation oncology recommendation was to proceed with a five-week course of radiation therapy post-operatively; however, radiation therapy was not completed in the immediate post-operative period to allow for adequate wound healing. The patient followed up with medical oncology service, which recommended surveillance imaging every three to six months and supportive care only given the poor performance status.

The patient then presented to the Emergency Department with vital signs notable for tachycardia with a heart rate of 107 bpm, hypertension with blood pressure of 150/72, and a new oxygen requirement of 4 L via nasal cannula. Physical examination was notable for decreased breath sounds bilaterally with mild bibasilar crackles, 1+ pitting edema bilaterally, and no notable calf tenderness.

**Figure 2:**
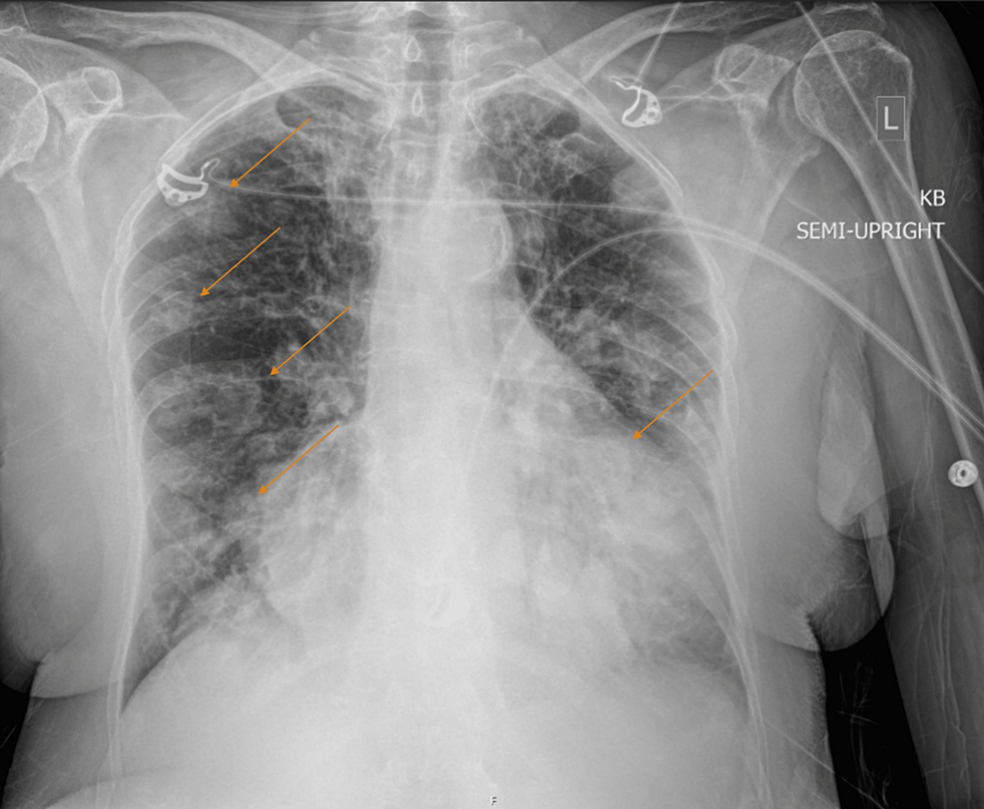
Multiple diffuse pulmonary masses bilaterally, consistent with metastatic disease

**Figure 3:**
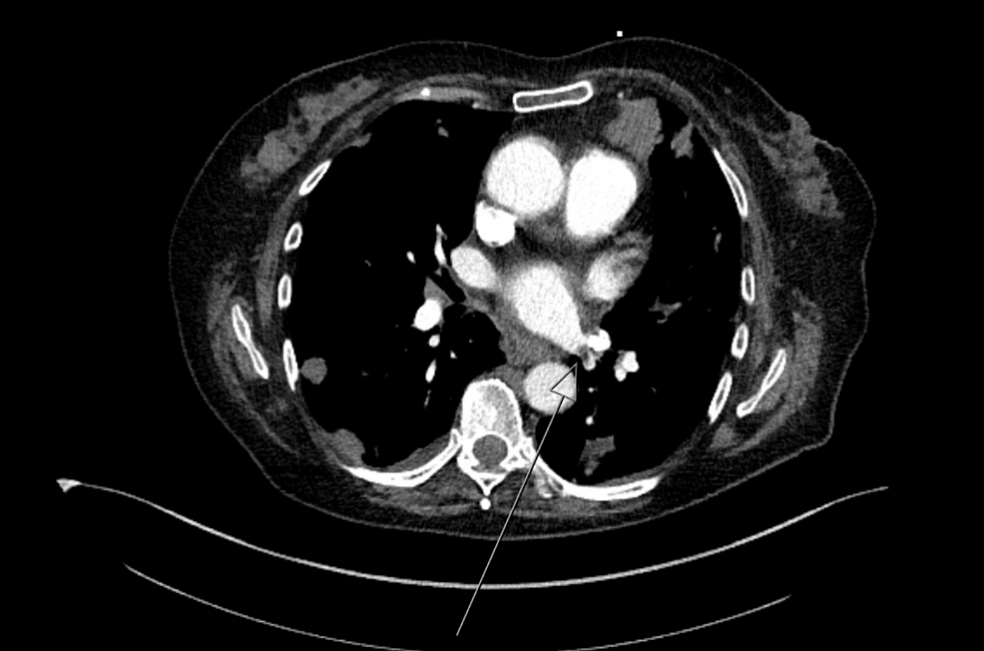
Filling defect in the inferior left pulmonary vein consistent with pulmonary vein thrombosis

Initial serologic evaluation demonstrated mild leukocytosis (WBC 11.3 K/uL), thrombocytosis (platelets 466 K/uL), and negative SARS-CoV-2 PCR. EKG demonstrated sinus tachycardia. Chest X-ray displayed multiple nodular-like opacities in bilateral lungs (Figure 2). CT angiography indicated a filling defect in the inferior left pulmonary vein consistent with PVT with redemonstration of bilateral lung masses, with the largest measuring 9.8 cm (Figure 3). Contrasted CT of the abdomen/pelvis demonstrated large soft tissue masses anterior to the right pelvis and right hip, measuring 9.3 cm and 3.8, cm respectively. The patient was started on full-dose anticoagulation with intravenous unfractionated heparin. Given the widespread metastasis and poor performance status, the patient was discharged with hospice care the following morning after extensive discussion with medical oncology.

## Discussion

PVT is a rare but potentially life-threatening condition with unclear incidence, as much of the literature stems from case reports [1-6]. In the published literature, PVT has most commonly been associated as a known complication of pulmonary lobectomy, lung transplant, radiofrequency ablation, and extracorporeal membrane oxygenation (ECMO), with rare reported cases of primary and secondary lung tumors. Other infrequent cases have been attributed to blunt chest trauma, sickle cell disease, and congenital narrowing of the pulmonary veins [7]. In regard to primary lung neoplasms, there are reports of PVT associated with bronchogenic carcinoma and small cell lung cancer, although current literature is limited [7]. While PVT has been associated with metastatic follicular thyroid carcinoma, renal cell carcinoma, small intestine mantle cell lymphoma, osteosarcoma, and liposarcoma in rare case reports, there is a lack of understanding regarding pathophysiology and mechanism of thrombosis in the pulmonary veins, with only proposed theories of potential extrinsic compression, endothelial disruption, hypercoagulability of malignancy, or direct tumor extension into the venous system [2]. Furthermore, there are no clear diagnostic criteria, gold standard imaging modality of choice, or therapeutic recommendation for the diagnosis and management of PVT in malignancy [3-7]. With this said, our case report of metastatic MFS as a cause of PVT provides another unique data point to the large disparity in current literature, with the hope that compilation of case reports can eventually lead to standard diagnostic and therapeutic recommendations.

Sarcoma is a neoplastic process that encompasses a broad spectrum of disease, generally accounting for less than 1% of all adult malignancies [8]. Liposarcoma and leiomyosarcoma are the most common, with MFS occurring in 20% of patients with sarcoma [9]. These tumors are generally slow-growing but have a high predisposition for local and distant recurrence, with increased propensity for lung metastases [10]. Unlike other sarcomas, MFS exhibits an infiltrative border that tends to extend into distant surrounding tissue via fascial planes, leading to microscopic tumor deposition highly prone to recurrence [11]. Interestingly, of the case reports of PVT caused by metastatic malignancy, the proportion of sarcoma cases (osteosarcoma, liposarcoma) seems to be oddly high in comparison with other types of malignancy with tendency for lung metastases. This raises the question of a predilection for PVT in sarcomatous malignancy, potentially related to the invasive nature or other characteristics that have yet to be described. Our case adds yet another type of sarcoma with distant metastasis to the current literature, which should raise questions for further research in this area.

PVT is a rare disease process, with overall incidence likely underestimated. Diagnosis poses a challenge, with multiple modalities such as CT angiogram, transthoracic echocardiogram (TTE), transesophageal echocardiogram (TEE), ventilation-perfusion (VQ) imaging, and bronchoscopy offering some clinical benefit depending on the location of the thrombus and clinical presentation [12]. As previously discussed, there is no diagnostic gold standard due to lack of data and rarity of PVT in malignancy. In our case, CT angiography effectively demonstrated multiple lung metastases as well as PVT, supporting this imaging modality in the diagnosis of PVT in malignancy. This is potentially advantageous over VQ imaging and TTE/TEE, as CT readily identifies parenchymal abnormalities potentially contributing to PVT. Given the limited data, there is no defined standard therapy regimen; however, it has been theorized that anticoagulant therapy with treatment of the underlying cause for PVT should be pursued for optimal therapy. Previous case reports have identified complications of cerebrovascular accident, right ventricular failure, pulmonary edema, allograft failure, and pulmonary infarction in patients with untreated or late presentation of PVT [2]. Again, a compilation of case reports is required to develop standard therapeutic recommendations.

## Conclusions

PVT in malignancy is a rare and life-threatening disease process only described in case reports without standard diagnostic or therapeutic recommendations given the lack of current data. Our case reinforces the need for further investigation into establishing a reliable diagnostic imaging modality, supporting the use of CT angiography in cases of known malignancy given the ability to identify parenchymal lesions that may be contributing to PVT. Furthermore, our case raises the question of a potential connection between different variants of sarcoma and PVT given an abnormally high ratio of case reports of sarcomatous malignancy and PVT. Finally, our case provides another unique data point to the large disparity in current literature, with the hope that compilation of case reports can eventually lead to standard diagnostic and therapeutic recommendations.
